# The impact of race and ethnicity on mortality and healthcare utilization in alcoholic hepatitis: a cross-sectional study

**DOI:** 10.1186/s12876-016-0544-y

**Published:** 2016-10-10

**Authors:** Folasade P. May, Vineet S. Rolston, Elliot B. Tapper, Ashwini Lakshmanan, Sammy Saab, Vinay Sundaram

**Affiliations:** 1 Division of Digestive Diseases, Department of Medicine, David Geffen School of Medicine at UCLA, 650 Charles E. Young Drive; Suite A2-125, Los Angeles, CA 90095-6900 USA; 2 Department of Health Policy and Management, UCLA Fielding School of Public Health, Los Angeles, CA USA; 3 Department of Medicine, Cedars-Sinai Medical Center, Los Angeles, CA USA; 4 Division of Gastroenterology, Beth Israel Deaconess Medical Center, Harvard Medical School, Boston, MA USA; 5 Department of Pediatrics, Center for Fetal and Neonatal Medicine, Children’s Hospital Los Angeles, Keck School of Medicine, University of Southern California, Los Angeles, CA USA; 6 Department of Surgery, David Geffen School of Medicine at UCLA, Los Angeles, CA USA; 7 Division of Gastroenterology and Hepatology and Comprehensive Transplant Center, Cedars-Sinai Medical Center, Los Angeles, CA USA

**Keywords:** Liver disease, Nationwide inpatient sample, Disparities, Healthcare utilization, Alcoholic hepatitis

## Abstract

**Background:**

Alcoholic Hepatitis (AH) is major source of alcohol-related mortality and health care expenditures in the United States. There is insufficient information regarding the role of race and ethnicity on healthcare utilization and outcomes for patients with AH. We aimed to determine whether there are racial/ethnic differences in resource utilization and inpatient mortality in patients hospitalized with AH.

**Methods:**

We analyzed data from the Nationwide Inpatient Sample (NIS), years 2008–2011. We calculated demographic, clinical, and healthcare utilization characteristics by race. We then performed logistic regression and generalized linear modeling with gamma distribution (log link), respectively, to determine predictors of inpatient morality and total hospital costs (THC).

**Results:**

We identified 11,304 AH patients from 2008 to 2011. Mean age was 47.0 years, and 62.1 % were male, 61.9 % were white, 9.8 % were black, and 9.7 % were Hispanic. Mean LOS was 6.3 days and significantly longer in whites (6.5 d) than both blacks (5.4 d) and Hispanics (5.9 d). In adjusted models, inpatient mortality was lower for blacks than for whites (adj. OR = 0.50; 95 % CI = 0.32–0.78). THC was significantly higher for Hispanics than whites (fold increase = 1.25; 95 % CI = 1.01–1.49).

**Conclusions:**

We identified differences in healthcare utilization and mortality by race/ethnicity. THC was significantly higher among Hispanics than for whites and blacks. We also demonstrated lower inpatient mortality in blacks compared to whites. These variations may implicate racial and ethnic differences in access to care, quality of care, severity of AH on presentation, or other factors.

**Electronic supplementary material:**

The online version of this article (doi:10.1186/s12876-016-0544-y) contains supplementary material, which is available to authorized users.

## Background

Alcoholic hepatitis (AH) is a major cause of alcohol-related morbidity and mortality in the United States (U.S.) [1, 2]. Characterized by acute liver inflammation in the setting of chronic alcohol use, AH carries a poor prognosis with a 30-day mortality as high as 30 %, depending on the severity of disease [3–5]. AH also poses significant burden on healthcare utilization and costs in the U.S. National data demonstrate that inpatient healthcare expenditures associated with AH may exceed spending for other chronic liver disease states, including hepatitis C (HCV) and hepatocellular carcinoma (HCC) [1, 6, 7]. As effective treatment is lacking for this condition, the mortality, healthcare costs, and utilization related to AH are unlikely to improve in the near future.

There is increasing emphasis on improving health equity and addressing healthcare disparities regarding race and ethnicity in the U.S. [8]. Current literature documents racial and ethnic disparities in mortality, prevalence, and healthcare utilization in several chronic liver disease states [9–13]. For example, blacks are less likely than white Americans to undergo screening for HCV and more likely than white Americans to die from HCV-related complications [11]. Studies have similarly demonstrated Hispanic Americans to have a greater prevalence of non-alcoholic fatty liver disease and an increased risk of developing HCC when compared to caucasians and blacks [9, 10, 12, 13].

While racial/ethnic variation in healthcare outcomes and utilization has been explored in many chronic liver disease states, there is a paucity of literature regarding the impact of these factors in relation to alcoholic hepatitis [14]. In this study, we aimed to determine whether there are racial and ethnic differences in inpatient mortality and health service utilization among patients hospitalized for AH in the U.S, using a large nationwide database.

## Methods

### Source of data

We performed a retrospective cross-sectional study using the Nationwide Inpatient Sample (NIS), years 2008–2011 [15]. The NIS is the largest publicly available inpatient database and includes a 20 % stratified sample of all U.S. inpatient discharges occurring in a given year from approximately 1000 non-federal U.S. hospitals in 42 to 47 states, depending on the year. Each hospital included in the NIS participates in the Agency for Healthcare Research and Quality Healthcare Cost and Utilization Project. Each discharge record from the NIS contains associated patient and hospital demographic information, primary and up to 24 secondary discharge diagnoses, and up to 15 procedural codes. The study protocol was approved as exempt from review by the institutional review board of Cedars-Sinai Medical Center.

### Study population

The study sample included inpatients age 18 years or older with a primary discharge diagnosis of AH. As per previously published studies utilizing administrative data to identify patients with AH, we used the International Classification of Diseases, 9th revision, Clinical Modification (ICD-9-CM) code 571.1 to identify our AH sample [2, 16].

### Variables

Our main outcomes were inpatient mortality, total hospital costs (THC), length of hospital stay (LOS), and number of procedures performed (NPR). THCs were inflated to 2011 values for years 2008, 2009, and 2010 using the consumer price index (CPI) [7]. We also accounted for inpatient procedures used in the management of AH, including nasogastric intubation (NGT), mechanical ventilation (MV), hemodialysis, esophagogastroduodenoscopy (EGD), paracentesis, liver biopsy, and liver transplantation.

Additional patient-level data included patient age, sex, race/ethnicity, socioeconomic status (SES), insurance type (Medicare, Medicaid, private, self-pay, other), comorbidity, source of admission (emergency room (ER), hospital transfer, other health-related facility, other non-health facility), and disposition at discharge (home or home with aid, other health facility, and other). For SES, we used income strata. For race, categories included white, black, Hispanic, and other (Asian, Pacific islander, Native American, or another race). Hospital characteristics in the analysis included hospital region (Northeast, South, Midwest, West), hospital type (teaching, nonteaching), and hospital setting (urban, rural).

We used the Deyo modification of the Charlson index as a proxy for patient comorbidity [17, 18]. We stratified the Charlson index into 3 groups to represent the degree of comorbidity: Mild (score = 0), moderate (score = 1–3), and severe (score > 3). In addition, we used the Baveno IV consensus criteria as a measure for severity of liver disease, where patients were categorized as stage 1 (no esophageal varices or ascites), stage 2 (esophageal varices, no ascites or bleeding), stage 3 (ascites, with or without esophageal varices), or stage 4 (history of gastrointestinal bleeding with or without ascites) [19]. Stage 1 and 2 disease represented compensated cirrhosis; stage 3 and 4 disease represented decompensated cirrhosis. As greater than 90 % of patients with biopsy-proven AH have underlying liver cirrhosis, this classification system is applicable to our study population [20].

We also accounted for known conditions that might impact AH outcomes, including ascites, HCV infection, gastrointestinal bleeding, hepatic encephalopathy, and bacterial infection (ICD-9 codes are provided in Additional file 1: Table S1) [21]. For infection, we created one variable that included all ICD-9-CM codes for bacterial infections potentially treated with antibiotics, similar to methodology used in previously published studies (ICD-9 codes are provided in Additional file 1: Table S1) [16, 22].

### Statistical analyses

We described patient descriptive and clinical characteristics as means with standard deviation or medians with interquartile ranges for Gaussian and non-Gaussian distributed variables, respectively. For crude statistical comparisons, we used the chi-square test for categorical variables, student’s t-test and one-way analysis of variance (ANOVA) with the Tukey-Kramer method to compare continuous variables, and the Wilcoxon-Rank sum test for non-Gaussian distributed variables, all with the Bonferroni correction for multiple comparisons. For THC, LOS and NPR, we computed regional comparisons of means by quantile regression.

Following descriptive analyses, we determined significant predictors of inpatient mortality and THC among patients admitted to U.S. hospitals with AH. We used multiple imputation for missing values for hospital admission source (19 % missing), discharge disposition (14 % missing), and race (14 % missing) using 20 imputed datasets. Other missing values were less than 5 % and were not imputed [23]. For mortality, we used logistic regression, controlling for patient demographic and clinical factors, to determine the impact of race/ethnicity on inpatient mortality among patients admitted with a primary diagnosis of AH. Second, we performed a generalized linear model (GLM) with gamma distribution and log link to examine the relationship between race/ethnicity and THC. GLM was felt appropriate in the present analysis given that THC is a heavily skewed, non-integer outcome. GLM compares the log of THC across groups while controlling for covariates in the model. Thus, the antilog of the parameters of the linear regression model can be interpreted as the fold increase in THC between groups being compared. For both regression models, covariates were selected based on significance in univariate analysis at the p <0.05 level or clinical experience and consistent prior data supporting their association. We used southern region as the reference geographic region as it was the region with the lowest mean value for THC based on unadjusted analysis.

Analyses were performed with STATA 13.1 software (Stata Corp, College Station, TX) with the appropriate survey estimation commands and strata weights provided in each NIS file. To represent national population estimates, we applied survey weights to the patient-level observations in the dataset. A p-value less than 0.05 on two-tailed testing was considered significant except in cases where the Bonferroni correction was indicated.

## Results

### Characteristics of study population

Table 1 provides demographic and clinical characteristics of the study sample of 11,304 AH patients, stratified by geographic region. Most of the patients were white (61.9 %), followed by black (9.8 %), and Hispanic (9.7 %). Mean age at admission was slightly lower in the Hispanic population (43.7 years) than in the white group (47.5 years; *p* < 0.01) and black group (48.2 y; *p* < 0.01). The majority of patients were white and male in each region.

**Table 1:** Demographic and clinical characteristics of patients hospitalized with alcoholic hepatitis from 2008–2011; *N* = 11,304^a,b^

Parameter	White *n* = 7019 (61.9 %)	Black *n* = 1103 (9.8 %)	Hispanic *n* = 1088 (9.7 %)	Other *n* = 548 (4.9 %)	Total^a^
Male Sex (%)	4093 (58.4)	665 (60.4)	894 (82.3)	378 (69.2)	7015 (62.1)
Mean Age, y (s.d)	47.5 (11.0)	48.2 (10.5)	43.7 (11.1)	44.8 (11.6)	47.0 (11.0)
Income (%)					
0–25 %	1428 (20.4)	607 (55.4)	366 (33.9)	177 (32.8)	3062 (27.2)
26–50 %	1845 (26.3)	223 (20.0)	248 (22.8)	102 (18.8)	2874 (25.4)
51–75 %	1837 (26.2)	147 (13.3)	250 (22.4)	110 (19.7)	2703 (23.8)
76–100 %	1707 (24.3)	78 (7.0)	157 (14.7)	117 (20.9)	2278 (20.1)
Payer (%)					
Medicare	972 (13.9)	191 (17.3)	103 (9.5)	58 (10.3)	1549 (13.7)
Medicaid	1345 (19.2)	338 (30.9)	301 (28.0)	163 (29.8)	2500 (22.2)
Private	2412 (34.4)	193 (17.3)	204 (18.6)	142 (25.9)	3441 (30.5)
Self-pay	1605 (22.7)	298 (26.9)	330 (30.4)	126 (23.1)	2703 (23.8)
Other	661 (9.3)	75 (6.9)	149 (13.4)	58 (10.9)	1063 (9.4)
Admission Source (%)					
Emergency room	1449 (20.3)	168 (15.1)	409 (37.2)	105 (18.7)	2238 (19.6)
Outside hospital	63 (0.9)	2 (0.2)	15 (1.4)	8 (1.4)	94 (0.8)
Non-health facility	16 (0.2)	2 (0.2)	0 (0.0)	2 (0.4)	21 (0.2)
Other health facility	477 (6.8)	103 (9.3)	48 (4.4)	32 (5.7)	693 (6.7)
Source unknown	5014 (71.8)	828 (75.2)	616 (57.1)	401 (73.9)	8258 (73.3)
Disposition (%)					
Home/Home with aid	4821 (68.8)	849 (77.3)	656 (60.8)	392 (71.8)	7927 (70.3)
Other inpatient facility	746 (10.7)	73 (6.7)	51 (4.7)	34 (6.3)	1115 (9.9)
Other	440 (6.3)	34 (3.1)	42 (3.9)	36 (6.7)	669 (5.9)
Unknown	1012 (14.3)	147 (12.9)	339 (30.5)	86 (15.3)	1593 (13.9)
Mean Charlson Index Score (s.d.)	1.7 (1.9)	1.4 (1.8)	1.6 (1.8)	1.6 (1.9)	1.6 (1.8)
Severity of Liver Disease (%)					
No cirrhosis	4913 (70.1)	869 (78.7)	773 (67.2)	377 (69.0)	8007 (70.9)
Compensated Cirrhosis	981 (13.9)	139 (12.7)	192 (17.8)	76 (13.8)	1608 (14.2)
Decompensated Cirrhosis	1125 (16.0)	95 (8.6)	163 (15.0)	95 (17.2)	1689 (14.9)
Known Hepatitis C (%)	556 (7.9)	121 (10.0)	87 (8.2)	35 (6.3)	921 (8.1)
Sepsis (%)	199 (2.9)	27 (2.4)	24 (2.2)	13 (2.4)	294 (2.6)
Gastrointestinal Bleed (%)	126 (1.8)	22 (2.0)	31 (2.9)	13 (2.3)	241 (2.1)
Ascites (%)	2030 (28.9)	165 (15.0)	251 (23.2)	161 (29.3)	3027 (26.7)
Hepatic Encephalopathy (%)	1066 (15.1)	90 (8.1)	126 (11.5)	65 (11.9)	1602 (14.1)
Any infection (%)	1792 (25.6)	226 (20.5)	222 (20.1)	128 (23.5)	2756 (24.3)
Hospital Type/Setting (%)					
Teaching	2947 (42.4)	646 (59.6)	597 (56.4)	289 (55.4)	5283 (47.4)
Urban	6209 (89.7)	994 (91.8)	1041 (97.0)	471 (89.8)	10014 (89.9)
U.S. Hospital Region (%)					
Northeast	1610 (23.5)	293 (27.4)	250 (23.5)	123 (22.4)	2314 (20.9)
Midwest	1118 (16.0)	180 (16.3)	76 (7.1)	82 (14.9)	2387 (21.2)
South	2614 (37.0)	527 (47.3)	313 (28.6)	146 (26.2)	3969 (34.8)
West	1677 (23.5)	103 (9.1)	449 (40.9)	197 (36.6)	2634 (23.1)

Other race refers to subjects with Asian, Pacific islander, Native American, or another race

*y* years

^a^Race/ethnicity was unknown for 1546 individuals not included in this table

^b^Data presented as unweighted n (weighted %) or mean (s.d.)

Weighted % in columns add to 100 %

Notably, over 50 % of blacks with AH were in the lowest quintile of income (55.4 %). In both blacks and Hispanics, Medicaid and self-pay (no insurance) were the most common insurance/payer status. There were also significant racial/ethnic differences in clinical characteristics. Blacks with AH were more likely than whites with AH to carry a diagnosis of HCV (10.0 % v 7.9 %; *p* < 0.01). Hispanics with AH were more likely than whites with AH to have a gastrointestinal bleed during hospitalization (2.9 % v. 1.8 %; *p* < 0.01). Decompensated cirrhosis was more common in whites with AH (16.0 %) and Hispanics with AH (15.0 %) than in blacks with AH (8.6 %).

### Outcomes and resource utilization

As demonstrated in Table 2, there were differences in utilization of hospital resources by race/ethnicity. Mean THC over the course of an inpatient hospitalization was significantly higher among Hispanics ($42,387) than among blacks ($33,359; *p* < 0.01). Mean LOS was longer among whites (6.5 d) compared to blacks (5.4 d) and Hispanics (5.9 d). The mean aggregate number of inpatient procedures performed was higher among whites than blacks (1.3 v. 1.1, *p* < 0.01) but similar between whites and Hispanics (1.3 v. 1.3, *p* = 0.16). Inpatient hospital mortality was lowest for blacks (2.3 %) and significantly lower in this racial group than for whites (4.5 %) (p<0.01) and Hispanics (3.9 %) (p<0.01). Only 4 liver transplants were reported in the entire sample. Given this low number, we did not compare this outcome by race/ethnicity.

**Table 2:** Healthcare utilization and mortality outcomes by race/ethnicity^a,b^

	White	Black	Hispanic	Other	Total	*P* value W v B	*P* value W v H	*P* value B v H
THC, $								
Mean	38,965.68	33,358.73	42,386.95	42,032.85	37,978.69	<0.01	0.09	<0.01
Median	23,399.00	20,585.54	25,530.00	24,094.00	23,020.00			
(IQR)	(41,903–13,915)	(37,782–11,944)	(43,287–15,110)	(44,952–13,444)	(40,607–13,501)			
LOS, d								
Mean	6.5	5.4	5.9	6.9	6.3	<0.01	0.01	0.04
Median	4	4	4	4	4			
(IQR)	(8–3)	(7–2)	(7–2)	(8–3)	(8–3)			
NPR								
Mean	1.3	1.1	1.3	1.5	1.3	<0.01	0.21	0.16
Median	1	0	1	1	1			
(IQR)	(2–0)	(1–0)	(2–0)	(2–0)	(2–0)			
Nasogastric tube (%)	26 (0.3)	3 (0.3)	5 (0.5)	2 (0.4)	43 (0.4)	0.67	0.60	0.48
Mechanical Ventilation (%)	241 (3.4)	34 (3.1)	34 (3.1)	24 (4.5)	385 (3.4)	0.54	0.62	0.93
Hemodialysis (%)	3 (<0.1)	0 (0)	1 (<0.1)	1 (0.2)	8 (<0.1)	--	0.53	--
Liver biopsy (%)	241 (3.4)	34 (3.2)	27 (2.5)	14 (3.1)	355 (3.2)	0.68	0.10	0.32
Paracentesis (%)	1259 (17.8)	103 (9.2)	154 (14.2)	94 (17.2)	1887 (16.6)	<0.001	<0.001	<0.001
EGD (%)	1111 (15.9)	153 (13.7)	161 (14.8)	86 (15.7)	1784 (15.8)	0.06	0.40	0.43
Deaths (%)	315 (4.5)	25 (2.3)	43 (3.9)	29 (5.3)	489 (4.3)	<0.001	0.38	0.03

Other race refers to subjects with Asian, Pacific islander, Native American, or another race

*W* white, *B* black, *H* hispanic, *THC* total hospital costs, *LOS* length of stay, *NPR* number of procedures, *IQR* interquartile range, *EGD* esophagogastroduodenoscopy

^a^Total hospital costs for 2008, 2009, and 2010 are inflated to 2011 values

^b^% values are weighted to national population estimates

-- there were no black subjects with hemodialysis

### Predictors of inpatient mortality

Table 3 provides significant predictors of inpatient mortality for hospitalized patients with AH in controlled models. Blacks were significantly less likely than whites to die while hospitalized for AH (adjusted OR = 0.50; 95 % CI = 0.32–0.78). Hispanics and whites had similar inpatient mortality. Additional significant predictors of inpatient mortality included increasing age, increasing Charlson index, severe cirrhosis, and greater than zero inpatient procedures performed during the current admission (Table 3).

**Table 3:** Predictors of inpatient mortality in patients with AH

Predictor	Died % (*n* = 489)	Odds Ratio (95 % CI)
Age	--	1.02 (1.01–1.03)***
Sex		
Male	322 (4.6)	Reference
Female	167 (3.9)	0.83 (0.67–1.01)
Race/Ethnicity		
White	315 (4.5)	Reference
Black	25 (2.3)	0.50 (0.32–0.78)**
Hispanic	43 (3.9)	0.89 (0.61–1.30)
Other	29 (5.3)	1.07 (0.70–1.66)
Income		
Lowest 25 %	139 (4.5)	1.21 (0.92–1.60)
25–50 %	122 (4.3)	Reference
50–75 %	117 (4.3)	1.06 (0.81–1.38)
75–100 %	97 (4.3)	0.87 (0.65–1.16)
Charlson comorbidity	--	1.41 (1.34–1.49)***
Severity of liver disease		
No cirrhosis	260 (3.3)	Reference
Stage 1–2	78 (4.9)	0.79 (0.58–1.07)
Stage 3–4	151 (9.0)	0.77 (0.59–0.99)*
Teaching Hospital		
No	234 (4.0)	0.96 (0.77–1.20)
Yes	245 (4.6)	Reference
Hospital setting		
Urban	432 (4.3)	Reference
Rural	47 (4.2)	0.89 (0.62–1.28)
Number of Procedures		
0	Reference	
1	54 (1.9)	1.53 (1.03–2.23)*
2	54 (3.8)	2.79 (1.83–4.25)***
> 2	332 (17.4)	13.3 (9.57–18.41)***

Data are presented as *n* (%) or odds ratios (95 % CI)

Final logistic regression model included survey weights and the following variables: sex, age, race/ethnicity, income, Charlson comorbidity score, severity of liver disease, teaching hospital status, hospital location, and number of inpatient procedures

Other race refers to subjects with Asian, Pacific islander, Native American, or another race

*CI* confidence interval

*P <0.05; **P <0.01; ***P <0.001

### Predictors of total hospital costs

Table 4 provides results for the predictor model for THC. When controlling for demographic and clinical factors, Hispanic ethnicity was associated with a 1.25-fold increase in THC when compared to white race. Thus, on average, a Hispanic patient admitted with AH had a THC 125 % that of a white patient admitted for the same duration of time. Despite this difference in whites and Hispanics, the THC in blacks was comparable to the THC in whites. Age, income, payer status, Charlson index, NPR, and hospitalization in the western U.S. were also significant predictors of inpatient costs.

**Table 4:** Predictors of inpatient THC in patients with AH

Predictor	Factor (fold increase)	95 % Confidence Interval
Age	0.99	0.98–0.99*
Sex		
Male	Reference	
Female	1.03	0.94–1.15
Race/Ethnicity		
White	Reference	
Black	1.24	0.96–1.60
Hispanic	1.25	1.01–1.49*
Other	0.94	0.79–1.13
Income		
Lowest 25 %	0.93	0.76–1.13
25–50 %	Reference	
50–75 %	1.07	0.92–1.25
75–100 %	1.18	1.03–1.34*
Payer		
Medicaid	0.85	0.73–0.98*
Medicare	0.94	0.81–1.09
Private	Reference	
Self-pay	0.75	0.62–0.91**
Other	0.84	0.72–0.99*
Length of Stay	1.02	1.02–1.03***
Charlson Index	1.04	1.02–1.07***
Severity of liver disease		
No cirrhosis	Reference	
Stage 1–2	1.12	0.97–1.31
Stage 3–4	1.01	0.89–1.15
Number of Procedures		
0	Reference	
1	1.27	1.20–1.34***
2	1.74	1.53–1.99***
> 2	2.97	2.72–3.25***
Hospital Region		
Northeast	Reference	
Midwest	1.06	0.85–1.33
South	0.88	0.70–1.11
West	1.32	1.02–1.71*
Teaching Hospital		
No	Reference	
Yes	1.10	0.94–1.28
Hospital setting		
Urban	1.28	1.11–1.48***
Rural	Reference	

Parameter coefficients are presented as a fold increase with 95 % CI

Final general linear model included survey weights and the following, variables: age, sex, race/ethnicity, income, payer, length of inpatient stay, Charlson comorbidity score, severity of liver disease, hospital region, teaching hospital status, hospital location, and number of inpatient procedures

Other race refers to subjects with Asian, Pacific islander, Native American, or another race

*CI* confidence interval

*P <0.05; **P <0.01; ***P <0.001

## Discussion

The cost of care and high resource utilization associated with AH is significant as demonstrated both in our study and in previous analyses [1]. However, there is currently little knowledge about variation in healthcare utilization and outcomes by race and ethnicity in this condition. This study of a nationally representative sample of hospitalized patients with AH contributes several novel findings regarding the relationships between race, outcomes and resource utilization. First, we demonstrated a higher average THC among Hispanics than whites and blacks. Secondly, we identified black race as an independent predictor for lower inpatient mortality compared to white race in patients hospitalized with AH.

Hispanic patients were shown to incur the highest charges in the nation, at 1.25 times higher than whites after adjusting for other factors including SES and payer source. These results are consistent with previous findings regarding other disease states that identified an association between Hispanic ethnicity and increased in-hospital service utilization, particularly for conditions characterized by organ failure and end-of-life [24–26]. Prior studies have further demonstrated that undocumented and uninsured Americans have poor access to timely medical care, particularly Hispanics who disproportionately live below the poverty line [27], and who may face exposure to unhealthy social and physical environments and lack of access to healthcare services [28–33]. Pervasive disparities in access to preventive services and primary care [29, 30, 32], as well as presentation with late-stage disease [34–36], likely contribute to increased resource utilization during inpatient hospitalization among Hispanics.

Differential use of the emergency room (ER) may also partially explain our findings of higher THC in Hispanics. We found that Hispanics utilized the ER as the source of admission significantly more frequently than whites or blacks. Consistent with other studies, ER-based referrals for inpatient hospitalizations are associated with higher THC [37]. Additionally, Hispanic patients who report alcohol use utilize ER services for the care of alcohol related consequences such as AH more commonly than non-Hispanics [38–40].

The finding that black patients with AH had lower adjusted mortality compared to other racial/ethnic groups is also notable. One possible explanation for this finding is that blacks do not have as severe a presentation of AH as compared to other race/ethnic groups. Previous findings from a single-center investigation demonstrated blacks (0 %) had a significantly lower proportion of patients presenting with severe AH, defined as discriminant function > 32, when compared to whites (39 %) and Hispanics (57 %) [14]. Although this previous study did not examine inpatient mortality, it can be inferred that the milder disease presentation among blacks would lead to a lower rate of death during hospitalization [14]. Our study, using a nationwide database, may support this hypothesis. Another explanation for lower inpatient mortality among blacks may be related to differences in provider care patterns between blacks and non-blacks. Studies in the literature have demonstrated lower inpatient mortality in blacks compared to whites for several conditions, which may be due to variation in quality of care or disease management and, possibly, earlier discharge and/or greater out of hospital mortality among blacks [41–43]. Our data source limited our ability to investigate these hypotheses; however, future studies should explore these potential explanations.

Our data must be interpreted in the context of the study’s limitations. First, NIS does not provide access to anthropometric or laboratory data. Such limitations might explain the relatively low rate of HCV seen in our AH cohort as HCVAb and HCV-RNA status may not have been known for all study subjects. It also limited us from distinguishing between non-severe AH and severe AH by calculating a discriminant function. However, we were able to account for comorbid medical conditions and severity of hepatic decompensation using the Charlson comorbidity score and Baveno IV measure. In doing so, we aimed to control for overall co-morbidity and liver disease severity, regardless of etiology. An additional limitation in our study was the inability to make conclusions about resource utilization and mortality for members of small race/ethnic groups (Asians, Pacific Islanders, Native Americans). As each of these groups was small, they were combined to create a heterogeneous ‘other race’ category. As a result, we were unable to analyze each of these groups separately. Lastly, as our study is a cross-sectional, observational study based on secondary data, we are unable to make conclusions about causation. These limitations are mitigated, however, by use of a large, nationally-representative database that enhances the generalizability of our results. Based on our findings, we suggest additional analyses to assess physician practice patterns in the management of AH patients, with attention to explanations for why racial and ethnic differences in mortality and resource utilization exist.

## Conclusions

In conclusion, our study demonstrated significant variation in inpatient mortality and inpatient costs between whites and racial/ethnic minorities hospitalized for AH in the U.S. Hispanics admitted for AH had similar mortality to but higher associated costs than whites admitted for AH. Blacks hospitalized for AH had lower inpatient mortality but similar inpatient costs to whites hospitalized for the same condition. We suspect that the differences in morality, cost, and resource utilization observed reflect multiple factors, including differences in access to care, comorbidity, AH severity at presentation, and quality of care delivered. Further investigation is warranted regarding the specific patient-, provider-, and system-level factors driving these findings to improve clinical outcomes, improve health equity, and curtail high healthcare costs.

## Additional file

Additional file 1: Table S1. International Classification of Diseases, 9^th^ Revision, Clinical Modification Code. This supplementary table include all ICD-9 codes for conditions included in the study analysis. (DOCX 100 kb)

## Abbreviations

AH: Alcoholic hepatitis
CPI: Consumer price index
EGD: Hemodialysis, esophagogastroduodenoscopy
ER: Emergency room
ICD-9 CM: International Classification of Diseases, 9th revision, Clinical Modification
IQR: Interquartile range
LOS: Length of stay
MV: Mechanical ventilation
NGT: Nasogastric intubation
NIS: Nationwide Inpatient Sample
NPR: Number of procedures
SES: Socioeconomic status
THC: Total hospital charges
US: United States

## References

[1] Jinjuvadia R, Liangpunsakul S, Consortium ftTRaEAHT. Trends in Alcoholic Hepatitis-related Hospitalizations, Financial Burden, and Mortality in the United States. J Clin Gastroenterol. 2015;49(6):506–11. 10.1097/MCG.0000000000000161 PMC4276725 25198164

[2] Liangpunsakul S (2011). Clinical characteristics and mortality of hospitalized alcoholic hepatitis patients in the United States. J Clin Gastroenterol.

[3] Basra S, Anand BS (2011). Definition, epidemiology and magnitude of alcoholic hepatitis. World J Hepatol.

[4] Maddrey WC, Boitnott JK, Bedine MS, Weber FL, Mezey E, White RI (1978). Corticosteroid therapy of alcoholic hepatitis. Gastroenterology.

[5] Thursz MR, Richardson P, Allison M, Austin A, Bowers M, Day CP, Downs N, Gleeson D, MacGilchrist A, Grant A (2015). Prednisolone or pentoxifylline for alcoholic hepatitis. N Engl J Med.

[6] Mishra A, Otgonsuren M, Venkatesan C, Afendy M, Erario M, Younossi ZM (2013). The inpatient economic and mortality impact of hepatocellular carcinoma from 2005 to 2009: analysis of the US nationwide inpatient sample. Liver Int.

[7] Younossi ZM, Otgonsuren M, Henry L, Arsalla Z, Stepnaova M, Mishra A, Venkatesan C, Hunt S (2015). Inpatient resource utilization, disease severity, mortality and insurance coverage for patients hospitalized for hepatitis C virus in the United States. J Viral Hepat.

[8] Institute of Medicine (IOM). Crossing the Quality Chasm. Crossing the Quality Chasm: A New Health System for the 21st Century. Washington, D.C: National Academy Press; 2001.

[9] Carrion AF, Ghanta R, Carrasquillo O, Martin P (2011). Chronic liver disease in the Hispanic population of the United States. Clin Gastroenterol Hepatol.

[10] El-Serag HB, Kramer J, Duan Z, Kanwal F (2014). Racial differences in the progression to cirrhosis and hepatocellular carcinoma in HCV-infected veterans. Am J Gastroenterol.

[11] Saab S, Jackson C, Nieto J, Francois F (2014). Hepatitis C in African Americans. Am J Gastroenterol.

[12] Saab S, Manne V, Nieto J, Schwimmer JB, Chalasani NP. Nonalcoholic Fatty Liver Disease in Latinos. Clin Gastroenterol Hepatol. 2016;14(1):5–12. 10.1016/j.cgh.2015.05.001 25976180

[13] Schneider AL, Lazo M, Selvin E, Clark JM (2014). Racial differences in nonalcoholic fatty liver disease in the U.S. population. Obesity.

[14] Levy RE, Catana AM, Durbin-Johnson B, Halsted CH, Medici V (2015). Ethnic differences in presentation and severity of alcoholic liver disease. Alcohol Clin Exp Res.

[15] Healthcare Cost and Utilization Project (HCUP). Content last reviewed September 2016. Rockville, MD: Agency for Healthcare Research and Quality. http://www.ahrq.gov/research/data/hcup/index.html. 21413206

[16] Sundaram V, May FP, Manne V, Saab S (2014). Effects of Clostridium difficile infection in patients with alcoholic hepatitis. Clin Gastroenterol Hepatol.

[17] Charlson ME, Pompei P, Ales KL, MacKenzie CR (1987). A new method of classifying prognostic comorbidity in longitudinal studies: development and validation. J Chronic Dis.

[18] Deyo RA, Cherkin DC, Ciol MA (1992). Adapting a clinical comorbidity index for use with ICD-9-CM administrative databases. J Clin Epidemiol.

[19] D'Amico G, Garcia-Tsao G, Pagliaro L (2006). Natural history and prognostic indicators of survival in cirrhosis: a systematic review of 118 studies. J Hepatol.

[20] Mathurin P, Louvet A, Duhamel A, Nahon P, Carbonell N, Boursier J, Anty R, Diaz E, Thabut D, Moirand R (2013). Prednisolone with vs without pentoxifylline and survival of patients with severe alcoholic hepatitis: a randomized clinical trial. JAMA.

[21] Louvet A, Wartel F, Castel H, Dharancy S, Hollebecque A, Canva-Delcambre V, Deltenre P, Mathurin P (2009). Infection in patients with severe alcoholic hepatitis treated with steroids: early response to therapy is the key factor. Gastroenterology.

[22] Ali M, Ananthakrishnan AN, Ahmad S, Kumar N, Kumar G, Saeian K (2012). Clostridium difficile infection in hospitalized liver transplant patients: a nationwide analysis. Liver Transplant.

[23] Little RJA, Rubin DB (2002). Statistical analysis with missing data.

[24] Barnato AE, Chang C-CH, Saynina O, Garber AM (2007). Influence of race on inpatient treatment intensity at the end of life. J Gen Intern Med.

[25] Hanchate A, Kronman AC, Young-Xu Y, Ash AS, Emanuel E (2009). Racial and ethnic differences in end-of-life costs: why do minorities cost more than whites?. Arch Intern Med.

[26] Propper B, Black JH, Schneider EB, Lum YW, Malas MB, Arnold MW, Abularrage CJ (2013). Hispanic ethnicity is associated with increased costs after carotid endarterectomy and carotid stenting in the United States. J Surg Res.

[27] Proctor D-WaB, United States Census Bureau UDoC (2014). Income and Poverty in the United States: 2013. Economics and Statistics Administration.

[28] Rodgers JT, Purnell JQ (2012). Healthcare navigation service in 2-1-1 San Diego: guiding individuals to the care they need. Am J Prev Med.

[29] Winkleby MA, Cubbin C (2003). Influence of individual and neighbourhood socioeconomic status on mortality among black, Mexican-American, and white women and men in the United States. J Epidemiol Community Health.

[30] Adler NE, Newman K (2002). Socioeconomic disparities in health: pathways and policies. Health Aff.

[31] Hanson MD, Chen E (2007). Socioeconomic status and health behaviors in adolescence: a review of the literature. J Behav Med.

[32] Phelan JC, Link BG, Tehranifar P (2010). Social conditions as fundamental causes of health inequalities: theory, evidence, and policy implications. J Health Soc Behav.

[33] Krueger PM, Chang VW (2008). Being poor and coping with stress: health behaviors and the risk of death. Am J Public Health.

[34] Bennett CL, Ferreira MR, Davis TC, Kaplan J, Weinberger M, Kuzel T, Seday MA, Sartor O (1998). Relation between literacy, race, and stage of presentation among low-income patients with prostate cancer. J Clin Oncol.

[35] Franks P, Fiscella K, Meldrum S (2005). Racial disparities in the content of primary care office visits. J Gen Intern Med.

[36] Lannin DR, Mathews HF, Mitchell J, Swanson MS, Swanson FH, Edwards MS (1998). Influence of socioeconomic and cultural factors on racial differences in late-stage presentation of breast cancer. JAMA.

[37] Chandwani HS, Strassels SA, Rascati KL, Lawson KA, Wilson JP (2013). Estimates of charges associated with emergency department and hospital inpatient care for opioid abuse-related events. J Pain Palliat Care Pharmacother.

[38] Bazargan-Hejazi S, Bazargan M, Hardin E, Bing EG (2005). Alcohol use and adherence to prescribed therapy among under-served Latino and African-American patients using emergency department services. Ethn Dis.

[39] Bazargan-Hejazi S, Bing E, Bazargan M, Der-Martirosian C, Hardin E, Bernstein J, Bernstein E (2005). Evaluation of a brief intervention in an inner-city emergency department. Ann Emerg Med.

[40] Lotfipour S, Cisneros V, Anderson CL, Roumani S, Hoonpongsimanont W, Weiss J, Chakravarthy B, Dykzeul B, Vaca F (2013). Assessment of alcohol use patterns among spanish-speaking patients. Subst Abus.

[41] Andrews RM, Moy E (2015). Racial differences in hospital mortality for medical and surgical admissions: variations by patient and hospital characteristics. Ethn Dis.

[42] Onukwugha E, Mullins CD (2007). Racial differences in hospital discharge disposition among stroke patients in Maryland. Med Decis Making.

[43] Volpp KG, Stone R, Lave JR, Jha AK, Pauly M, Klusaritz H, Chen H, Cen L, Brucker N, Polsky D (2007). Is thirty-day hospital mortality really lower for black veterans compared with white veterans?. Health Serv Res.

