# Supplementary material for: The impact of race and ethnicity on mortality and healthcare utilization in alcoholic hepatitis: a cross-sectional study
Source: BMC Gastroenterol. 2016 Oct 10;16:129. doi: 10.1186/s12876-016-0544-y (PMC5057210; doi:10.1186/s12876-016-0544-y)
Supplement: Additional file 1: Table S1. — International Classification of Diseases, 9th Revision, Clinical Modification Code. This supplementary table include all ICD-9 codes for conditions included in the study analysis. (DOCX 100 kb) [file 12876_2016_544_MOESM1_ESM.docx]

**Supplementary Table**

| **Disease Codes** | **International Classification of Diseases, 9^th^ Revision, Clinical Modification Codes** |
| --- | --- |
| Alcoholic hepatitis | 571.1 |
| Cirrhosis | 571.2; 571.5; 571.6 |
| Hepatitis C | 070.54; 070.44; 070.70; 070.71 |
| Sepsis | 995.91; 995.92 |
| Gastrointestinal bleed | 578; 578.9; 578.0; 578.1; 456.0; 456.1; 456.2; 456.8; 456.21 |
| Esophageal varices | 456.0; 456.1; 456.2; 456.8; 456.21 |
| Ascites | 789.5; 789.59; |
| Hepatic encephalopathy | 572.2; 348.30 |
| Any Infection | Septicemia: 038.0, 038.10, 038.11, 038.19, 038.2, 038.3, 038.40, 038.41, 038.42, 038.43, 038.44, 038.49, 038.8, and 038.9  Systemic inflammatory response syndrome caused by infectious process without organ dysfunction: 995.91  Bacteremia: 790.7  Septic shock: 785.52  Systemic inflammatory response syndrome due to infectious process with organ dysfunction: 995.92  Intestinal infection due to clostridium difficile: 008.45  Fungal:  Disseminated fungal infection: 117.9  Disseminated candidal infection: 112.5  Candidal endocarditis: 112.81  Fungal endocarditis: 115.04, 115.14, 115.94  Candidal meningitis: 112.83  Fungal meningitis: 114.2, 115.01, 115.11, 115.91  Fungal pneumonia: 115.05, 115.15, 115.95  Rhinosporidiosis: 117.0  Zygomycosis: 117.7  Aspergillosis: 117.3  Other fungal: 484.6, 484.7, 321.0, 321.1,  Disseminated fungal infection: 117.9  Disseminated candidal infection: 112.5  Candidal endocarditis: 112.81  Fungal endocarditis: 115.04, 115.14, 115.94,  Meningococcal septicemia: 036.2  Waterhouse-Friderichsen syndrome: 036.3  Gram-positive Staphylococcus:  Toxic shock syndrome: 040.82  Scalded skin syndrome: 695.81  Pneumonia: 482.40– 482.42, 482.49  Meningitis: 320.3  Septicemia: 038.10, 038.11, 038.19  Unspecified site: 041.10– 19  Streptococcus: 038.0, 041.00– 09, 482.30– 2, 482.39, 320.2  Erysipelas: 035  Strep throat/scarlet fever: 034.0, 034.1  Rheumatic fever: 390, 391.0– 2, 391.8– 9, 392  Pneumococcal: 038.2, 320.1, 481, 567.1  Gram negative  Escherichia coli : 041.4, 038.42,482.82  Helicobacter influenza : 041.5, 038.41, 482.2, 320.0  Proteus: 041.6  Klebsiella: 482.0  Legionella: 482.84  Pseudomonas: 041.7, 038.43, 482.1  Serratea: 038.44  Other gram negative: 041.85, 038.40, 038.49, 482.83, 320.82  Anaerobes  Pneumonia: 482.81  Meningitis: 320.81  Bacteroides: 041.82  Clostridium perfringens: 041.83  Unspecified: 041.84  Septicemia: 038.3  Central nervous system abscess: 324.0, 324, 1, 324.9  Abscess of pharynx: 478.21, 478.22, 478.24  Peritonsillar abscess: 475  Empyema: 510.0, 510.9  Lung abscess: 513.0, 513.1  Peritonitis: 567.0, 567.1, 567.21, 567.22, 567.23, 567.29  Anal and rectal abscess: 566  Abdominal abscess: 567.31, 567.38, 567.39, 567.81, 567.9  Intestinal abscess: 569.5  Perforation of intestine: 569.83  Abscess of liver: 572.0  Portal pyemia: 572.1  Orchitis with abscess: 604.0  Other cellulitis or abscess: 682.0-9  Cellulitis, finger/toe: 681.00, 681.01, 681.02, 681.10, 681.11, 681.9  Acute lymphadenitis: 683  Pyogenic arthritis: 711.00-09  Osteomyelitis: 730.00-09  Postoperative infection: 998.51, 998.59  Infection or inflammation of device/graft, infection from Foley catheters: 996.64  Infection from any central line: 999.31  Infectious complication of medical care not otherwise classified: 999.3  Pyelonephritis: 590.10, 590.11, 590.2, 590.3, 590.80, 590.81, 590.9  Other cellulitis or abscess: 682.0– 9  Bacterial meningitis: 320.0– 3, 320.7, 320.81, 320.82, 320.89, 320.9  Meningitis due to other organism: 321.0– 4, 321.8  Phlebitis of intracranial sinus: 325  Acute or subacute endocarditis: 421.0, 421.1, 421.9  Thrombophlebitis: 451.0, 451.11, 451.19, 451.2, 451.81, 4510.82, 451.83, 451.84, 451.89, 451.9  Acute sinusitis: 461.0-3, 461.8, 461.9  Acute pharyngitis: 462  Acute tonsillitis: 463  Acute URI: 465.0, 465.8, 465.9  Bronchitis: 466.0, 466.11, 466.19  Abscess of pharynx: 478.21, 478.22, 478.24  Peritonsillar abscess: 475  Pneumococcal pneumonia: 481  Other bacterial pneumonia: 482.0– 2, 482.30– 32, 482.39, 482.40, 482.41, 482.49, 482.81-84, 482.89, 484.1, 484.3, 484.5, 484.6, 484.7, 484.8, 483.0, 483.1, 483.8  Bronchopneumonia: organism not specified 485  Pneumonia: organism not specified 486  Acute COPD exacerbation: 491.21, 491.22  Bronchiectasis: 494.1  Empyema: 510.0, 510.9  Lung abscess: 513.0, 513.1  Acute appendicitis: 540.0, 540.1, 540.9  Appendicitis not: 541  Other appendicitis: 542  Diverticulitis of small intestine without hemorrhage: 562.01  Diverticulitis of small intestine with hemorrhage: 562.03  Diverticulitis of colon without hemorrhage: 562.11  Diverticulitis of colon with hemorrhage: 562.13  Acute cholecystitis: 575.0  Peritonitis: 567.0, 567.1, 567.21, 567.22, 567.23, 567.29  Anal and rectal abscess: 566  Abdominal abscess: 567.31, 567.38, 567.39, 567.81, 567.9  Intestinal abscess: 569.5  Perforation of intestine: 569.83  Abscess of liver: 572.0  Portal pyemia: 572.1  Cystitis: 595.0  Urethritis/urethral syndrome: 597.0  Urinary tract infection not otherwise specified: 599.0  Pyelo: 590.10, 590.11, 590.2, 590.3, 590.80, 590.81, 590.9  Prostatic inflammation: 601.0, 601.2, 601.9  Female pelvic inflammation disease: 614.0, 614.2, 614.3, 614.5, 614.9  Uterine inflammatory disease: 615.0, 615.9  Other female genital inflammation: 616.3, 616.4  Orchitis with abscess: 604.0  Other cellulitis or abscess: 682.0-9  Cellulitis, fi nger/toe: 681.00, 681.01, 681.02, 681.10, 681.11, 681.9  Acute lymphadenitis: 683  Pyogenic arthritis: 711.00-09  Osteomyelitis: 730.00-09  Postoperative infection: 998.51, 998.59  Infection or inflammation of device/graft, infection from Foley catheters: 996.64  Infection from any central line: 999.31  Infectious complication of medical care not otherwise classified: 999.3 |
| **Procedure Codes** | **International Classification of Diseases, 9^th^ Revision, Clinical Modification Codes** |
| Nasogastric Intubation | 96.06, 96.07 |
| Mechanical ventilation | 96.0, 96.7, 96.04 |
| Hemodialysis | 39.95, V45.11, V56.0 |
| Esophagogastroduodenoscopy (EGD) | 45.13, 45.16, V82.9 |
| Paracentesis | 54.91 |
| Liver Biopsy | 50.1, 50.11, 50.12, 50.13, 50.14 |
| Liver Transplantation | V42.7, 50.5, 50.51, 50.59 |
| Thoracentesis | 34.91 |
| Chest Tube | 34.04 |
| Urinary Catheter | 57.94 |
| Central Venous Catheter | 38.93, 38.95, 86.93, 89.64, 89.66, 89.67, 89.68 |
| Parenteral Nutrition | 99.15 |
